# COVID-19 in Nepal: Lower Than Expected Incidence and Mortality

**DOI:** 10.1177/1010539520940884

**Published:** 2020-07-07

**Authors:** Krishna Prasad Pathak

**Affiliations:** 1 Nepal Open University, Lalitpur, Nepal

COVID-19, which originated in Wuhan, China, in December 2019, is highly infectious and has spread rapidly around the world.^1^ The World Health Organization^2^ declared COVID-19 to be a pandemic and has now infected more than 7 million people. Nepal has had 2300 cases and 9 deaths (as of May 17, 2020).^3^ The first case was recorded in Nepal on January 24 in a 31-year-old Nepali student who recently returned from Wuhan in China, a city where a large number of Nepali and Indian students go to study medicine and other advanced courses. The 31-year-old student had returned from Wuhan, China, on January 5. He had visited a hospital for respiratory problems and was admitted on January 13. Negative results for COVID-19 were given by consecutive follow-up tests on January 29 and 31.^4^

Nepal has a relative lack of health services and testing facilities, made worse by shortages of personal protecting equipment (PPE). It is interesting to speculate on why COVID-19 has a lower incidence in many lower income countries, such as Nepal, when compared with Europe and North America. Full nationwide lockdown, isolation of cases and contacts, contact tracing, social distancing, and quarantine of new arrivals are the best ways of preventing the spread of infection. One hypothesis is that countries with high rates of Bacillus Calmette-Guerin vaccination may have lower rates. Nepal has a younger population than other countries with few larger cities that are likely to facilitate rapid spread. The population of 28 million has a median age of 23 years, only 4% are older than 65 years of age, and 80% live in rural locations. These factors may at least partly explain our low rates of infection.

The pandemic of COVID-19 is still evolving, and it is too early to predict the outcome of the current outbreak. It may become a seasonal pathogen, like influenza, which might return every winter with seasonal outbreaks.

We believe that Nepal needs a more robust safety net for workers with paid sick leave, universal health insurance, or health care system. The government needs to increase funding for local health departments, begin planning for future epidemics, and be prepared to bolster the economy by supporting consumer spending in the midst of a serious outbreak.

COVID-19 is a serious health challenge for Nepal, but so far, the number of deaths has been lower than was anticipated.
